# *N*-{4-[(2-Meth­oxy­phen­yl)sulfamo­yl]phen­yl}acetamide

**DOI:** 10.1107/S1600536811000432

**Published:** 2011-01-08

**Authors:** Saba Ahmad, Muhammad Akhyar Farrukh, Fahim Ashraf Qureshi, Ahmad Adnan, Mehmet Akkurt

**Affiliations:** a Department of Chemistry, Government College University, Lahore 54000, Pakistan; b Department of Physics, Faculty of Sciences, Erciyes University, 38039 Kayseri, Turkey

## Abstract

In the title compound, C_15_H_16_N_2_O_4_S, the S atom has a distorted tetra­hedral geometry [maximum deviation: O—S—O = 118.25 (7)°]. The two aromatic rings make a dihedral angle of 62.67 (10)° with each other. An intra­molecular N—H⋯O hydrogen bond forms an *S*(6) ring motif. In the crystal, mol­ecules form centrosymmetric dimers *via* pairwise N—H⋯O inter­actions, forming an *R*_2_^2^(8) ring motif, and these dimers are connected by N—H⋯O hydrogen bonds, generating a three-dimensional network. Furthermore, a weak C—H⋯π inter­action helps to reinforce the crystal structure. The O atom in the acetamide group is disordered over two positions with major and minor occupancies of 0.52 (5) and 0.48 (5), respectively.

## Related literature

For background and the biological activity of sulfonamide and its derivatives, see: Korolkovas (1988 ▶); Mandell & Sande (1992 ▶); Pandya *et al.* (2003 ▶); Supuran & Scozzafava (2001 ▶). For related structures, see: Aziz-ur-Rehman *et al.* (2010*a* ▶,**b* ▶,c* ▶); Khan *et al.* (2010 ▶). For hydrogen-bond motifs, see: Bernstein *et al.* (1995 ▶). 
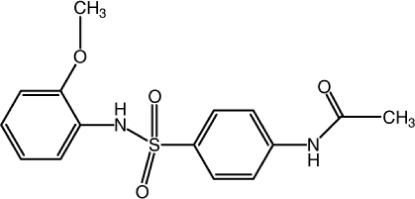


## Experimental

### 

#### Crystal data

C_15_H_16_N_2_O_4_S
*M* *_r_* = 320.37
Orthorhombic, 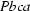

*a* = 15.7277 (4) Å
*b* = 11.8351 (3) Å
*c* = 16.5247 (4) Å
*V* = 3075.89 (13) Å^3^
*Z* = 8
Mo *K*α radiation
μ = 0.23 mm^−1^
*T* = 296 K
0.24 × 0.18 × 0.09 mm

#### Data collection

Bruker APEXII CCD diffractometer
15586 measured reflections
3788 independent reflections
2800 reflections with *I* > 2σ(*I*)
*R*_int_ = 0.035

#### Refinement

*R*[*F* ^2^ > 2σ(*F* ^2^)] = 0.042
*wR*(*F* ^2^) = 0.118
*S* = 1.03
3788 reflections
220 parameters
2 restraints
H atoms treated by a mixture of independent and constrained refinement
Δρ_max_ = 0.35 e Å^−3^
Δρ_min_ = −0.28 e Å^−3^

### 

Data collection: *APEX2* (Bruker, 2007 ▶); cell refinement: *SAINT* (Bruker, 2007 ▶); data reduction: *SAINT*; program(s) used to solve structure: *SIR97* (Altomare *et al.*, 1999 ▶); program(s) used to refine structure: *SHELXL97* (Sheldrick, 2008 ▶); molecular graphics: *ORTEP-3 for Windows* (Farrugia, 1997 ▶); software used to prepare material for publication: *WinGX* (Farrugia, 1999 ▶) and *PLATON* (Spek, 2009 ▶).

## Supplementary Material

Crystal structure: contains datablocks global, I. DOI: 10.1107/S1600536811000432/hg2782sup1.cif

Structure factors: contains datablocks I. DOI: 10.1107/S1600536811000432/hg2782Isup2.hkl

Additional supplementary materials:  crystallographic information; 3D view; checkCIF report

## Figures and Tables

**Table 1:** Hydrogen-bond geometry (Å, °) *Cg*2 is the centroid of the C9–C14 ring.

*D*—H⋯*A*	*D*—H	H⋯*A*	*D*⋯*A*	*D*—H⋯*A*
N1—H*N*1⋯O3^i^	0.811 (19)	2.191 (19)	2.995 (2)	171.0 (19)
N2—H*N*2⋯O4	0.823 (18)	2.359 (19)	2.6551 (19)	102.0 (15)
N2—H*N*2⋯O2^ii^	0.823 (18)	2.259 (18)	3.0482 (18)	160.6 (18)
C5—H5⋯*Cg*2^iii^	0.93	2.90	3.715 (2)	148

Symmetry codes: (i) 
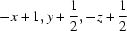
; (ii) 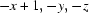; (iii) 
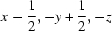
.

**Fig. 1.**
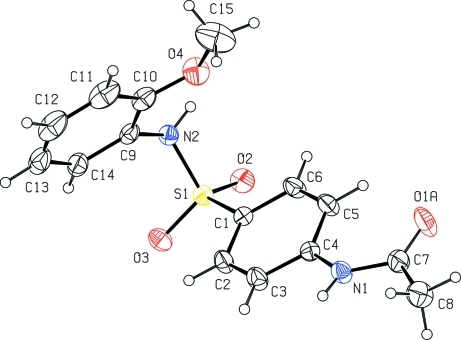
View of the title compound with the atom numbering scheme. Displacement ellipsoids for non-H atoms are drawn at the 30% probability level. Only the major component of the disorder is shown.

**Fig. 2.**
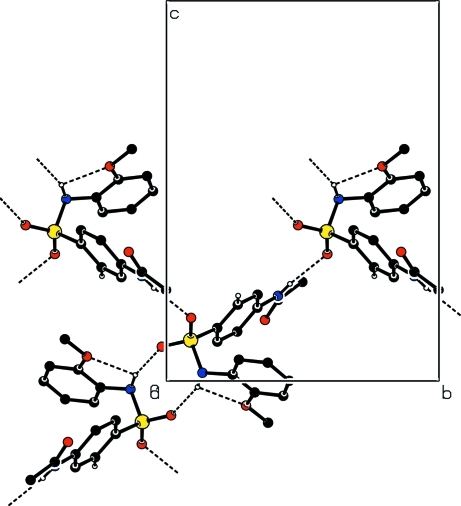
View of the dimeric N—H···O interactions between the molecules and the other hydrogen bonding interactions in the unit cell. H atoms not involved in hydrogen bonds (dashed lines) have been omitted for clarity and only the major component of the disorder is shown.

## supplementary crystallographic information

### Comment

Sulfonamides are very important because of their antibacterial and enzyme inhibitor properties as well as their extensive use in medicine (Pandya *et al.*, 2003). Sulfonamides also exhibit antimicrobial activity (Korolkovas, 1988; Mandell & Sande, 1992) and have their properties to inhibit the growth of tumor cells (Supuran & Scozzafava, 2001). As a contribution to a structural study of sulfonamide derivatives (Khan *et al.*, 2010; Aziz-ur-Rehman *et al.*, 2010*a*,*b*,*c*), we report here the title compound, *N*-{4-[(2-methoxyphenyl)sulfamoyl]phenyl}acetamide, (I).

In the title molecule (I), (Fig. 1), the S atom has a distorted tetrahedral geometry [maximum deviation: O1—S1—O2 = 118.25 (7) °]. The molecule is twisted at the S atom, with a C1—S1—N2—C9 torsion angle of 56.88 (14) °. The dihedral angle formed between the benzene (C1–C6) and phenyl (C9–C14) rings in (I) is 62.67 (10)°.

An intramolecular N2—HN2···O4 hydrogen bond contribute to the stabilization of the molecular conformation, forming an S(6) ring motif (Table 1; Bernstein *et al.*, 1995). In the crystal structure, the molecules of (I) are dimerized due to the intermolecular N—H···O hydrogen bonding (Table 1, Fig. 2) forming an *R*_2_^2^(8) ring motif (Table 1; Bernstein *et al.*, 1995) and these dimers are connected by N—H···O hydrogen bonds, generating a three-dimensional network (Table 1, Fig. 2).

### Experimental

5 mmol of 2-methoxyaniline was dissolved in 20 ml of distilled water then 5 mmol of 4-acetamidobenzenesulfonyl chloride was addedd. The reaction mixture was stirred for about 2–3 h while the pH of the reaction mixture was maintained between 8–10 using 3% Na_2_CO_3_. The reaction was monitored by TLC. The precipitate formed was filtered, washed with distilled water, dried and recrystallized by using methanol.

### Refinement

The amino H atoms are located in a difference Fourier map and refined freely. The remaining H atoms were positioned geometrically with C—H = 0.93 for aromatic H and C—H = 0.96 Å for methyl H and allowed to ride on their parent atoms, with *U*_iso_(H) = 1.2*U*_eq_(C_aromatic_) or 1.5*U*_eq_(C_methyl_). The oxygen atom in the acetamide group is disordered over two positions with a major and minor occupancy of 0.52 (5) and 0.48 (5), respectively.

### Crystal data

**Table:**

C_15_H_16_N_2_O_4_S	*F*(000) = 1344
*M**_r_* = 320.37	*D*_x_ = 1.384 Mg m^−^^3^
Orthorhombic, *P**b**c**a*	Mo *K*α radiation, λ = 0.71073 Å
Hall symbol: -P 2ac 2ab	Cell parameters from 4792 reflections
*a* = 15.7277 (4) Å	θ = 2.5–27.6°
*b* = 11.8351 (3) Å	µ = 0.23 mm^−^^1^
*c* = 16.5247 (4) Å	*T* = 296 K
*V* = 3075.89 (13) Å^3^	Block, colourless
*Z* = 8	0.24 × 0.18 × 0.09 mm

### Data collection

**Table:**

Bruker APEXII CCD diffractometer	2800 reflections with *I* > 2σ(*I*)
Radiation source: sealed tube	*R*_int_ = 0.035
graphite	θ_max_ = 28.3°, θ_min_ = 3.4°
φ and ω scans	*h* = −17→20
15586 measured reflections	*k* = −14→15
3788 independent reflections	*l* = −22→19

### Refinement

**Table:**

Refinement on *F*^2^	Primary atom site location: structure-invariant direct methods
Least-squares matrix: full	Secondary atom site location: difference Fourier map
*R*[*F*^2^ > 2σ(*F*^2^)] = 0.042	Hydrogen site location: inferred from neighbouring sites
*wR*(*F*^2^) = 0.118	H atoms treated by a mixture of independent and constrained refinement
*S* = 1.03	*w* = 1/[σ^2^(*F*_o_^2^) + (0.0648*P*)^2^ + 0.4113*P*] where *P* = (*F*_o_^2^ + 2*F*_c_^2^)/3
3788 reflections	(Δ/σ)_max_ < 0.001
220 parameters	Δρ_max_ = 0.35 e Å^−^^3^
2 restraints	Δρ_min_ = −0.28 e Å^−^^3^

### Special details

**Table:**

Geometry. Bond distances, angles *etc*. have been calculated using the rounded fractional coordinates. All su's are estimated from the variances of the (full) variance-covariance matrix. The cell e.s.d.'s are taken into account in the estimation of distances, angles and torsion angles
Refinement. Refinement on *F*^2^ for ALL reflections except those flagged by the user for potential systematic errors. Weighted *R*-factors *wR* and all goodnesses of fit *S* are based on *F*^2^, conventional *R*-factors *R* are based on *F*, with *F* set to zero for negative *F*^2^. The observed criterion of *F*^2^ > σ(*F*^2^) is used only for calculating -*R*-factor-obs *etc*. and is not relevant to the choice of reflections for refinement. *R*-factors based on *F*^2^ are statistically about twice as large as those based on *F*, and *R*-factors based on ALL data will be even larger.

### Fractional atomic coordinates and isotropic or equivalent isotropic displacement parameters (Å^2^)

**Table:**

	*x*	*y*	*z*	*U*_iso_*/*U*_eq_	Occ. (<1)
S1	0.56431 (2)	0.08868 (3)	0.10743 (2)	0.0342 (1)	
O1A	0.1823 (9)	0.3615 (19)	0.1605 (11)	0.071 (3)	0.52 (5)
O2	0.52415 (8)	−0.01701 (9)	0.09026 (8)	0.0464 (4)	
O3	0.63163 (8)	0.09054 (10)	0.16600 (8)	0.0464 (4)	
O4	0.52707 (9)	0.29215 (11)	−0.06451 (9)	0.0603 (5)	
N1	0.30275 (9)	0.40934 (13)	0.22401 (10)	0.0439 (5)	
N2	0.60326 (9)	0.13213 (11)	0.02165 (8)	0.0354 (4)	
C1	0.48747 (10)	0.18646 (13)	0.13933 (9)	0.0336 (5)	
C2	0.50585 (11)	0.26178 (14)	0.20091 (11)	0.0436 (5)	
C3	0.44366 (11)	0.33454 (15)	0.22693 (12)	0.0472 (6)	
C4	0.36276 (10)	0.33370 (13)	0.19280 (10)	0.0367 (5)	
C5	0.34562 (11)	0.25968 (15)	0.12988 (11)	0.0462 (6)	
C6	0.40841 (12)	0.18647 (16)	0.10356 (11)	0.0453 (6)	
C7	0.21664 (12)	0.40990 (17)	0.21306 (12)	0.0521 (6)	
C8	0.17075 (13)	0.49861 (19)	0.26052 (14)	0.0639 (8)	
C9	0.64089 (10)	0.24249 (13)	0.01705 (10)	0.0374 (5)	
C10	0.60126 (13)	0.32392 (14)	−0.03012 (12)	0.0474 (6)	
C11	0.64036 (18)	0.42861 (17)	−0.03904 (15)	0.0696 (9)	
C12	0.71585 (18)	0.4507 (2)	0.00053 (17)	0.0790 (10)	
C13	0.75310 (15)	0.3717 (2)	0.04807 (14)	0.0687 (8)	
C14	0.71651 (12)	0.26592 (17)	0.05603 (12)	0.0511 (6)	
C15	0.4802 (2)	0.3729 (2)	−0.10987 (18)	0.0967 (13)	
O1B	0.1793 (9)	0.326 (2)	0.1809 (15)	0.083 (3)	0.48 (5)
HN1	0.3247 (12)	0.4533 (16)	0.2553 (11)	0.055 (6)*	
HN2	0.5717 (11)	0.1143 (16)	−0.0160 (11)	0.047 (6)*	
H3	0.45580	0.38550	0.26820	0.0570*	
H5	0.29230	0.25920	0.10550	0.0550*	
H6	0.39710	0.13680	0.06130	0.0540*	
H8A	0.20100	0.56890	0.25660	0.0960*	
H8B	0.16740	0.47600	0.31620	0.0960*	
H8C	0.11440	0.50790	0.23920	0.0960*	
H10D	0.46860	0.43760	−0.07660	0.1450*	
H10E	0.42760	0.33990	−0.12750	0.1450*	
H10F	0.51290	0.39580	−0.15620	0.1450*	
H11	0.61570	0.48360	−0.07160	0.0840*	
H12	0.74150	0.52090	−0.00560	0.0950*	
H13	0.80320	0.38860	0.07540	0.0820*	
H14	0.74270	0.21090	0.08750	0.0610*	
H2	0.55960	0.26310	0.22430	0.0520*	

### Atomic displacement parameters (Å^2^)

**Table:**

	*U*^11^	*U*^22^	*U*^33^	*U*^12^	*U*^13^	*U*^23^
S1	0.0362 (2)	0.0288 (2)	0.0376 (2)	0.0018 (2)	−0.0030 (2)	0.0035 (2)
O1A	0.037 (4)	0.095 (7)	0.082 (5)	0.008 (3)	−0.019 (3)	−0.040 (5)
O2	0.0568 (8)	0.0291 (6)	0.0532 (7)	−0.0046 (5)	−0.0027 (6)	0.0050 (5)
O3	0.0457 (7)	0.0455 (7)	0.0480 (7)	0.0078 (5)	−0.0120 (6)	0.0032 (5)
O4	0.0614 (9)	0.0490 (8)	0.0704 (10)	0.0066 (7)	−0.0070 (7)	0.0153 (7)
N1	0.0383 (8)	0.0469 (9)	0.0464 (8)	0.0019 (6)	−0.0032 (6)	−0.0141 (7)
N2	0.0367 (7)	0.0320 (7)	0.0374 (7)	−0.0013 (6)	0.0001 (6)	−0.0036 (6)
C1	0.0340 (8)	0.0343 (8)	0.0325 (8)	−0.0002 (6)	0.0015 (6)	0.0006 (6)
C2	0.0345 (8)	0.0467 (9)	0.0497 (10)	−0.0020 (7)	−0.0064 (7)	−0.0103 (8)
C3	0.0407 (9)	0.0509 (10)	0.0499 (10)	−0.0020 (8)	−0.0048 (8)	−0.0192 (8)
C4	0.0359 (8)	0.0394 (8)	0.0348 (8)	−0.0011 (7)	0.0014 (6)	−0.0026 (6)
C5	0.0377 (9)	0.0560 (11)	0.0449 (9)	0.0068 (8)	−0.0111 (7)	−0.0119 (8)
C6	0.0431 (9)	0.0524 (11)	0.0403 (9)	0.0058 (8)	−0.0092 (7)	−0.0143 (8)
C7	0.0390 (9)	0.0689 (13)	0.0483 (10)	0.0050 (9)	0.0014 (8)	−0.0119 (9)
C8	0.0514 (12)	0.0760 (14)	0.0644 (13)	0.0240 (11)	−0.0038 (10)	−0.0101 (11)
C9	0.0393 (8)	0.0345 (8)	0.0384 (8)	−0.0053 (7)	0.0122 (7)	−0.0070 (7)
C10	0.0567 (11)	0.0363 (9)	0.0493 (10)	−0.0026 (8)	0.0133 (9)	−0.0009 (8)
C11	0.1000 (19)	0.0387 (11)	0.0702 (14)	−0.0098 (11)	0.0275 (13)	0.0028 (10)
C12	0.102 (2)	0.0542 (13)	0.0809 (17)	−0.0404 (14)	0.0384 (16)	−0.0221 (13)
C13	0.0634 (14)	0.0780 (15)	0.0647 (13)	−0.0357 (12)	0.0228 (11)	−0.0303 (13)
C14	0.0432 (10)	0.0601 (12)	0.0499 (10)	−0.0109 (9)	0.0097 (8)	−0.0145 (9)
C15	0.128 (3)	0.0751 (16)	0.087 (2)	0.0394 (18)	−0.0316 (18)	0.0058 (14)
O1B	0.050 (3)	0.091 (7)	0.109 (7)	−0.021 (4)	0.023 (4)	−0.047 (5)

### Geometric parameters (Å, °)

**Table:**

S1—O2	1.4297 (12)	C9—C14	1.381 (2)
S1—O3	1.4347 (13)	C9—C10	1.387 (2)
S1—N2	1.6276 (14)	C10—C11	1.391 (3)
S1—C1	1.7543 (16)	C11—C12	1.380 (4)
O1A—C7	1.172 (19)	C12—C13	1.355 (4)
O1B—C7	1.27 (2)	C13—C14	1.384 (3)
O4—C15	1.421 (3)	C2—H2	0.9300
O4—C10	1.351 (2)	C3—H3	0.9300
N1—C4	1.399 (2)	C5—H5	0.9300
N1—C7	1.366 (2)	C6—H6	0.9300
N2—C9	1.436 (2)	C8—H8A	0.9600
N1—HN1	0.811 (19)	C8—H8B	0.9600
N2—HN2	0.823 (18)	C8—H8C	0.9600
C1—C2	1.383 (2)	C11—H11	0.9300
C1—C6	1.377 (2)	C12—H12	0.9300
C2—C3	1.372 (2)	C13—H13	0.9300
C3—C4	1.392 (2)	C14—H14	0.9300
C4—C5	1.386 (2)	C15—H10D	0.9600
C5—C6	1.384 (3)	C15—H10E	0.9600
C7—C8	1.496 (3)	C15—H10F	0.9600
			
S1···H14	3.1700	C8···H10E^ix^	3.0800
S1···H3^i^	3.1800	C11···H10F	2.8100
S1···H13^ii^	3.2000	C11···H10D	2.7700
O1A···C5	2.882 (16)	C11···H10D^xii^	3.0200
O1A···C10^iii^	3.33 (2)	C13···H8B^xiii^	2.8900
O1A···N2^iii^	3.257 (18)	C13···H5^vii^	3.0400
O1A···C9^iii^	3.248 (19)	C13···H6^vii^	2.9000
O1B···C5	2.858 (16)	C14···H5^vii^	2.9400
O1B···C10^iii^	3.30 (2)	C15···H11	2.5800
O2···N2^iv^	3.0482 (18)	HN1···H8A	2.3800
O2···O2^iv^	3.1045 (19)	HN1···O3^vi^	2.191 (19)
O3···N1^i^	2.995 (2)	HN1···H3	2.2200
O3···C14	3.065 (2)	H2···O1B^xiii^	2.5600
O4···N2	2.6551 (19)	H2···O3	2.5300
O1A···H5	2.3000	HN2···O4	2.359 (19)
O1B···H2^v^	2.5600	HN2···O2^iv^	2.259 (18)
O1B···H5	2.3100	H3···S1^vi^	3.1800
O2···H3^i^	2.6300	H3···O2^vi^	2.6300
O2···HN2^iv^	2.259 (18)	H3···HN1	2.2200
O2···H6	2.7500	H5···O1A	2.3000
O3···H14	2.6000	H5···C7	2.7800
O3···H2	2.5300	H5···O1B	2.3100
O3···HN1^i^	2.191 (19)	H5···C13^iii^	3.0400
O4···HN2	2.359 (19)	H5···C14^iii^	2.9400
N1···O3^vi^	2.995 (2)	H6···C13^iii^	2.9000
N2···O1A^vii^	3.257 (18)	H6···O2	2.7500
N2···O2^iv^	3.0482 (18)	H8A···HN1	2.3800
N2···O4	2.6551 (19)	H8B···C13^v^	2.8900
N2···H12^ii^	2.8100	H10D···C11^xii^	3.0200
C2···C15^viii^	3.533 (3)	H10D···C11	2.7700
C5···O1A	2.882 (16)	H10D···H11	2.3800
C5···O1B	2.858 (16)	H10E···C8^xi^	3.0800
C6···C13^iii^	3.566 (3)	H10F···H11	2.3800
C8···C15^ix^	3.541 (4)	H10F···C2^x^	3.0100
C9···O1A^vii^	3.248 (19)	H10F···C11	2.8100
C10···O1A^vii^	3.33 (2)	H11···C15	2.5800
C10···O1B^vii^	3.30 (2)	H11···H10D	2.3800
C13···C6^vii^	3.566 (3)	H11···H10F	2.3800
C14···O3	3.065 (2)	H11···C4^xii^	2.9700
C15···C2^x^	3.533 (3)	H12···N2^xiv^	2.8100
C15···C8^xi^	3.541 (4)	H13···S1^xiv^	3.2000
C2···H10F^viii^	3.0100	H14···S1	3.1700
C4···H11^xii^	2.9700	H14···O3	2.6000
C7···H5	2.7800		
			
O2—S1—O3	118.25 (7)	C9—C10—C11	118.66 (19)
O2—S1—N2	105.66 (7)	C10—C11—C12	119.9 (2)
O2—S1—C1	109.42 (7)	C11—C12—C13	121.1 (2)
O3—S1—N2	107.75 (7)	C12—C13—C14	120.0 (2)
O3—S1—C1	107.19 (7)	C9—C14—C13	119.70 (18)
N2—S1—C1	108.21 (7)	C1—C2—H2	120.00
C10—O4—C15	118.87 (16)	C3—C2—H2	120.00
C4—N1—C7	128.52 (16)	C2—C3—H3	119.00
S1—N2—C9	119.25 (11)	C4—C3—H3	119.00
C4—N1—HN1	111.0 (13)	C4—C5—H5	120.00
C7—N1—HN1	120.2 (13)	C6—C5—H5	120.00
S1—N2—HN2	110.5 (13)	C1—C6—H6	120.00
C9—N2—HN2	116.2 (13)	C5—C6—H6	120.00
S1—C1—C6	119.55 (13)	C7—C8—H8A	109.00
C2—C1—C6	120.30 (15)	C7—C8—H8B	109.00
S1—C1—C2	120.14 (12)	C7—C8—H8C	109.00
C1—C2—C3	119.07 (16)	H8A—C8—H8B	109.00
C2—C3—C4	121.34 (17)	H8A—C8—H8C	109.00
N1—C4—C3	117.56 (15)	H8B—C8—H8C	109.00
C3—C4—C5	119.10 (15)	C10—C11—H11	120.00
N1—C4—C5	123.34 (15)	C12—C11—H11	120.00
C4—C5—C6	119.52 (16)	C11—C12—H12	119.00
C1—C6—C5	120.64 (17)	C13—C12—H12	120.00
O1B—C7—C8	123.0 (8)	C12—C13—H13	120.00
O1A—C7—N1	123.5 (8)	C14—C13—H13	120.00
O1A—C7—C8	120.6 (9)	C9—C14—H14	120.00
N1—C7—C8	114.34 (17)	C13—C14—H14	120.00
O1B—C7—N1	120.7 (8)	O4—C15—H10D	109.00
N2—C9—C14	120.87 (15)	O4—C15—H10E	109.00
C10—C9—C14	120.64 (16)	O4—C15—H10F	109.00
N2—C9—C10	118.45 (15)	H10D—C15—H10E	110.00
O4—C10—C9	115.53 (15)	H10D—C15—H10F	109.00
O4—C10—C11	125.81 (18)	H10E—C15—H10F	110.00
			
O2—S1—N2—C9	173.98 (12)	S1—C1—C6—C5	177.38 (14)
O3—S1—N2—C9	−58.72 (13)	C6—C1—C2—C3	1.4 (3)
C1—S1—N2—C9	56.88 (14)	C1—C2—C3—C4	0.2 (3)
O2—S1—C1—C2	141.15 (13)	C2—C3—C4—C5	−1.7 (3)
O3—S1—C1—C2	11.77 (15)	C2—C3—C4—N1	178.84 (16)
N2—S1—C1—C2	−104.20 (14)	C3—C4—C5—C6	1.5 (3)
O2—S1—C1—C6	−37.82 (16)	N1—C4—C5—C6	−179.03 (16)
O3—S1—C1—C6	−167.20 (13)	C4—C5—C6—C1	0.1 (3)
N2—S1—C1—C6	76.84 (15)	N2—C9—C10—C11	−175.85 (18)
C15—O4—C10—C9	176.41 (19)	C14—C9—C10—O4	−179.16 (17)
C15—O4—C10—C11	−4.5 (3)	N2—C9—C14—C13	177.52 (17)
C7—N1—C4—C5	15.0 (3)	C10—C9—C14—C13	0.1 (3)
C4—N1—C7—C8	175.71 (18)	C14—C9—C10—C11	1.7 (3)
C4—N1—C7—O1A	−18.4 (13)	N2—C9—C10—O4	3.3 (2)
C7—N1—C4—C3	−165.53 (18)	O4—C10—C11—C12	179.1 (2)
S1—N2—C9—C14	68.93 (19)	C9—C10—C11—C12	−1.8 (3)
S1—N2—C9—C10	−113.58 (16)	C10—C11—C12—C13	0.2 (4)
C2—C1—C6—C5	−1.6 (3)	C11—C12—C13—C14	1.6 (4)
S1—C1—C2—C3	−177.52 (13)	C12—C13—C14—C9	−1.7 (3)

Symmetry codes: (i) −*x*+1, *y*−1/2, −*z*+1/2; (ii) −*x*+3/2, *y*−1/2, *z*; (iii) *x*−1/2, −*y*+1/2, −*z*; (iv) −*x*+1, −*y*, −*z*; (v) *x*−1/2, *y*, −*z*+1/2; (vi) −*x*+1, *y*+1/2, −*z*+1/2; (vii) *x*+1/2, −*y*+1/2, −*z*; (viii) *x*, −*y*+1/2, *z*+1/2; (ix) −*x*+1/2, −*y*+1, *z*+1/2; (x) *x*, −*y*+1/2, *z*−1/2; (xi) −*x*+1/2, −*y*+1, *z*−1/2; (xii) −*x*+1, −*y*+1, −*z*; (xiii) *x*+1/2, *y*, −*z*+1/2; (xiv) −*x*+3/2, *y*+1/2, *z*.

### Hydrogen-bond geometry (Å, °)

**Table:**

Cg2 is the centroid of the C9–C14 ring.

**Table:**

*D*—H···*A*	*D*—H	H···*A*	*D*···*A*	*D*—H···*A*
N1—HN1···O3^vi^	0.811 (19)	2.191 (19)	2.995 (2)	171.0 (19)
N2—HN2···O4	0.823 (18)	2.359 (19)	2.6551 (19)	102.0 (15)
N2—HN2···O2^iv^	0.823 (18)	2.259 (18)	3.0482 (18)	160.6 (18)
C2—H2···O3	0.93	2.53	2.890 (2)	104
C5—H5···O1A	0.93	2.30	2.882 (16)	120
C5—H5···Cg2^iii^	0.93	2.90	3.715 (2)	148

Symmetry codes: (vi) −*x*+1, *y*+1/2, −*z*+1/2; (iv) −*x*+1, −*y*, −*z*; (iii) *x*−1/2, −*y*+1/2, −*z*.
